# Risk stratification model based on estimated dose of radiation to immune cells and radiotherapy-related nadir lymphocyte count for predicting the efficacy of consolidation immunotherapy in stage III non-small cell lung cancer

**DOI:** 10.3389/fimmu.2026.1734341

**Published:** 2026-07-09

**Authors:** Wenlu Chen, Yu Liang, Qing Hou, Xin Cao, Anqi Zhao, Baixue Wu, Yuying Zhou, Wenbo Zhang, Meng Zhao, Ningning Yao, Feng Li, Jianchun Duan, Shuangping Zhang, Ning Li, Jianzhong Cao

**Affiliations:** 1 Department of Radiotherapy, Shanxi Province Cancer Hospital/Shanxi Hospital Affiliated to Cancer Hospital, Chinese Academy of Medical Sciences/Cancer Hospital Affiliated to Shanxi Medical University, Taiyuan, China; 2 Shanxi Key Laboratory of Tumor Organoids, Shanxi Province Cancer Hospital/Shanxi Hospital Affiliated to Cancer Hospital, Chinese Academy of Medical Sciences/Cancer Hospital Affiliated to Shanxi Medical University, Taiyuan, China; 3 State Key Laboratory of Molecular Oncology, Beijing Key Laboratory, CAMS Key Laboratory of Translational Research on Lung Cancer, Department of Medical Oncology, Cancer Hospital, Chinese Academy of Medical Sciences, Beijing, China; 4 Department of Respiratory Medicine, Shanxi Province Cancer Hospital, Shanxi Hospital Affiliated to Cancer Hospital, Chinese Academy of Medical Sciences, Cancer Hospital Affiliated to Shanxi Medical University, Taiyuan, China; 5 Department of Thoracic Surgery, Shanxi Province Cancer Hospital/Shanxi Hospital Affiliated to Cancer Hospital, Chinese Academy of Medical Sciences/Cancer Hospital Affiliated to Shanxi Medical University, Taiyuan, China

**Keywords:** chemoradiotherapy, estimated dose of radiation to immune cells, immunotherapy, nadir absolute lymphocyte count, risk stratification, stage III non-small cell lung cancer

## Abstract

**Purpose:**

This study aimed to evaluate the prognostic value of the estimated dose of radiation to immune cells (EDRIC) and the radiotherapy-related nadir lymphocyte count (RT-NLC), and to establish a risk stratification model for predicting outcomes in patients with unresectable stage III non-small cell lung cancer (NSCLC) treated with definitive chemoradiotherapy (CRT) and immunotherapy.

**Materials and methods:**

We retrospectively analyzed 136 patients treated between May 2019 and November 2023. EDRIC and RT-NLC were dichotomized at median values. Survival analysis was performed using Kaplan–Meier and Cox regression. High-risk patients were defined as having EDRIC ≥ 6.75 Gy and RT-NLC < 0.54×10^9^/L.

**Results:**

EDRIC inversely correlated with RT-NLC (r = –0.38, *P* < 0.001). Lower EDRIC was associated with significantly improved median overall survival (OS: 49.7 vs. 38.1 months, *P* = 0.015), progression-free survival (PFS: 29.7 vs. 17.3 months, *P* = 0.006), locoregional relapse-free survival (LRFS: 32.4 vs. 19.8 months, *P* = 0.004), and distant metastasis-free survival (DMFS: 44.8 vs. 24.0 months, *P* = 0.001). On multivariable analysis, low EDRIC independently predicted improved OS (HR = 0.51), PFS (HR = 0.56), LRFS (HR = 0.53), and DMFS (HR = 0.42). High RT-NLC group was significantly prolonged median DMFS (44.8 vs 26.8 months, *P* = 0.012). High-risk patients demonstrated inferior survival (*P* < 0.05) but derived significant benefit from consolidation immunotherapy, with improved OS (HR = 0.37, *P* = 0.041), PFS (HR = 0.44, *P* = 0.034), and DMFS (HR = 0.33, *P* = 0.011).

**Conclusions:**

EDRIC and RT-NLC are significant prognostic biomarkers in unresectable stage III NSCLC. A risk model integrating these parameters effectively identifies high-risk patients who obtain substantial survival benefit, particularly in distant metastasis control, from consolidation immunotherapy, supporting personalized treatment strategies. Prospective validation is warranted.

## 1 Introduction

Lung cancer continues to be the most common cause of cancer-related mortality worldwide, with approximately 1.8 million deaths projected annually (1). Non-small cell lung cancer (NSCLC) constitutes 80%–85% of all lung cancer cases, and about 30% of those are diagnosed at stage III (2). For patients with unresectable stage III NSCLC, definitive chemoradiotherapy (CRT) has long been the standard of care; however, outcomes remain suboptimal, with reported 5-year progression-free survival (PFS) and overall survival (OS) rates of only 18.3% and 32.1%, respectively (3).

In recent years, immune checkpoint inhibitors (ICIs), particularly as consolidation therapy following CRT, have significantly improved outcomes in this population (4). Radiotherapy (RT) exhibits dual immunomodulatory effects when combined with ICIs. On one hand, it can enhance antitumor immunity through mechanisms such as increased tumor antigen release, improved antigen presentation, and induction of immunogenic cell death. On the other hand, RT also exerts immunosuppressive effects, notably through the induction of radiotherapy-related lymphopenia (RT-NLC). Given the high radiosensitivity of lymphocytes, RT-NLC is commonly observed during CRT and has been consistently correlated with an unfavorable prognosis across multiple malignancies (5–8). Severe RT-NLC may not only compromise antitumor immune surveillance but also diminish the efficacy of subsequent immunotherapy, posing a significant challenge to realizing the synergistic potential of RT and ICIs.

To address this issue, the estimated dose of radiation to immune cells (EDRIC) has been proposed as a novel dosimetric parameter, aiming to quantify the systemic immunologic impact of radiotherapy (9–11). The EDRIC model incorporates the median lung dose (MLD), median heart dose (MHD), and median body dose (MBD) to estimate the radiation exposure of circulating immune cells. Previous studies, including a secondary analysis of the RTOG 0617 trial, have demonstrated that higher EDRIC was significantly associated with worse OS and locoregional control (12). Subsequent investigations in NSCLC populations have reaffirmed the independent prognostic value of EDRIC, suggesting that lower EDRIC correlates with improved clinical outcomes (9, 13).

EDRIC is a predictive dosimetric parameter derived from radiotherapy planning that reflects the potential extent of radiation-induced immune cell damage, whereas RT-NLC is a dynamic biological marker representing the actual degree of immune injury and immune reserve during treatment. Although both parameters provide complementary insights, each has inherent limitations when used independently: EDRIC does not account for interindividual variability in immune sensitivity, while RT-NLC may be influenced by non-radiotherapy-related factors such as chemotherapy and underlying comorbidities. Therefore, EDRIC may be interpreted as reflecting exposure risk, whereas RT-NLC represents the corresponding immune outcome; integrating these parameters may mitigate the limitations of each single indicator and enable a more comprehensive assessment of immune status and long-term prognosis.

However, most existing studies have focused on patients treated with CRT alone, without incorporating immunotherapy. As immunotherapy has now become an integral component of standard treatment, it remains unclear whether EDRIC retains its prognostic value in the context of combined-modality therapy. Furthermore, the relationship between RT-NLC, a real-time biomarker of immune status, and EDRIC, a planning-based predictive metric, remains unclear. It is also unknown whether these two indicators can be integrated into a clinically useful risk stratification model to predict outcomes and guide immunotherapy decisions.

Therefore, this study aimed to retrospectively analyze patients with unresectable stage III NSCLC treated with definitive CRT and ICIs. We sought to investigate the correlation between EDRIC and RT-NLC, and to evaluate their individual and combined prognostic value for survival outcomes. Our goal is to provide new evidence to inform risk stratification and clinical decision-making for this patient population.

## 2 Materials and methods

### 2.1 Patient population

This single-center retrospective study included consecutive patients with stage III NSCLC who received definitive CRT combined with immunotherapy between May 2019 and November 2023. The study protocol was approved by the institutional ethics committee (Approval No: KY2025006). Eligibility criteria included age ≥18 years, histologically or cytologically confirmed NSCLC, and stage IIIA–IIIC disease according to the 8th edition AJCC TNM staging system. Unresectable status was defined in accordance with the National Comprehensive Cancer Network (NCCN) clinical practice guidelines (14) and was confirmed by a multidisciplinary team (MDT) including thoracic surgeons, radiation oncologists, and medical oncologists. Only patients without known driver gene mutations were included. All patients completed definitive thoracic radiotherapy and received at least two cycles of platinum-based chemotherapy and immunotherapy. Exclusion criteria included: (1) history of other malignancies or previous thoracic radiotherapy; (2) prior molecular targeted therapy; or (3) severe comorbidities affecting treatment evaluation or follow-up.

### 2.2 Clinical data collection

We collected baseline clinicopathological and treatment-related data from the electronic medical records, including age, sex, smoking history, Eastern Cooperative Oncology Group performance status (ECOG PS), TNM stage, chemotherapy regimen (concurrent or sequential), number of chemotherapy/immunotherapy cycles, and glucocorticoid use. We obtained radiotherapy parameters from the treatment planning system: fractionation scheme (conventional vs. hypofractionation), RT technique [intensity-modulated radiotherapy (IMRT) or volumetric modulated arc therapy (VMAT)], prescription dose, gross tumour volume (GTV), planning target volume (PTV), median lung dose (MLD), median heart dose (MHD), and median body dose (MBD). The baseline absolute lymphocyte count (ALC) was measured before treatment, with the lower normal limit set at 1.1 × 10^9^/L. RT-NLC was defined as the nadir value recorded during radiotherapy based on weekly blood counts (≥3 measurements).

### 2.3 Treatment and follow-up

All patients received definitive CRT combined with immunotherapy, administered either as induction chemoimmunotherapy followed by CRT (with or without consolidation), or as CRT followed by consolidation immunotherapy. ICIs included PD-1/PD-L1 inhibitors such as pembrolizumab, camrelizumab, sintilimab, tislelizumab, nivolumab, sugemalimab, penpulimab, durvalumab, or atezolizumab. Chemotherapy regimens were selected based on histology: paclitaxel plus carboplatin or cisplatin for squamous cell carcinoma, and pemetrexed plus carboplatin or cisplatin for non-squamous subtypes. Radiotherapy was delivered using IMRT/VMAT following 4D-CT simulation, with prescribed doses ranging from 60–66 Gy in 30–33 fractions. Treatment plans were generated using the Philips Pinnacle system, ensuring that at least 95% of the PTV received 100% of the prescribed dose. Target volumes were delineated as follows: primary tumour volume (GTVp) was contoured on the simulation CT; nodal tumour volume (GTVn) encompassed lymph nodes with a short-axis diameter ≥1 cm or with increased FDG uptake on PET-CT; the clinical target volume (CTV) was defined as GTV (GTVp + GTVn) plus a 0.5 cm margin; and the PTV was defined as the CTV plus a 0.5–1.0 cm margin to account for set-up uncertainties and organ motion. Post-treatment follow-up was performed every 3 months during the first year, every 6 months during the second year, and annually thereafter. Survival data were collected through outpatient visits and telephone follow-up.

### 2.4 Calculation of EDRIC

EDRIC was computed based on the modified Ladbury model, which was originally proposed by Jin et al. (15) and further refined by Ladbury et al. (9). This formula has been validated in previous studies, further improving its credibility. It integrating MLD, MHD, and MBD to estimate the radiation exposure of circulating immune cells using the formula:


$$\text{EDRIC}=0.12\times \text{MLD}+0.08\times \text{MHD}+[0.45+0.35\times 0.85{\left(\frac{\#\text{of fractions}}{45}\right)}^{\frac{1}{2}}]\times \text{MBD}$$

All dosimetric parameters were extracted from the RT planning system.

### 2.5 Statistical analysis

Statistical analyses were performed using R software (version 4.2.3). The primary endpoint was OS, and secondary endpoints included PFS, LRFS, and distant metastasis-free survival (DMFS). OS was defined as the time from treatment initiation to death from any cause or last follow-up; PFS as the time from treatment initiation to progression or death; LRFS as the time from treatment initiation to locoregional progression or death; and DMFS as the time from treatment initiation to distant metastasis or death. Median values dichotomized continuous variables (16): patients were classified into a high EDRIC group (≥6.75 Gy) or a low EDRIC group (<6.75 Gy), and into a low RT-NLC group (<0.54 × 10^9^/L) or a high RT-NLC group (≥0.54 × 10^9^/L). Patients with EDRIC ≥ 6.75Gy and RT-NLC < 0.54 × 10^9^/L were defined as the high-risk group, while the remainder were classified as the low-risk group. Continuous variables are expressed as mean ± standard deviation (SD) and compared using the independent samples t-test. Categorical variables were compared using the χ² test or Fisher’s exact test as appropriate. Spearman’s rank correlation was used to assess the association between EDRIC and RT-NLC. Survival curves were generated using the Kaplan–Meier method and compared with log-rank tests. Cox proportional hazards models were used for univariable and multivariable analyses. Variables with *P* < 0.1 in univariable analysis were included in the multivariable model, which was optimized by minimizing the Akaike information criterion (AIC). To address potential immortal time bias, landmark analyses were performed using predefined time points of 6 and 12 months. Hazard ratios (HRs) and 95% confidence intervals (CIs) were reported. A two-sided *P* < 0.05 was considered statistically significant.

## 3 Results

A total of 136 patients with unresectable stage III NSCLC met the inclusion criteria. The baseline clinical characteristics are summarized in **Table 1**. The cohort was predominantly male (91.9%), with most patients exhibiting an ECOG performance status of 0–1 (89.7%). Among the participants, 53.7% were under 65 years of age, and 46.3% were 65 or older. According to the 8th edition AJCC staging system, 46.3% were classified as stage IIIA, 39.0% as stage IIIB, and 14.7% as stage IIIC. The majority (86.0%) had a history of smoking. Concurrent chemoradiotherapy was administered to 29.4% of patients, while 70.6% received sequential treatment. Most patients received ≤6 cycles of immunotherapy (71.3%) and chemotherapy (70.6%). Immunotherapy was administered as consolidation in 52.2% of patients, as induction in 33.1%, and as both induction and consolidation in 14.7%. At baseline, the mean ALC was 1.80 ± 0.56 ×10^9^/L. Glucocorticoid (GC) use was observed in 25.7% of patients, while 74.3% did not receive GC.

**Table 1:** Baseline characteristics of patients (values presented as n [%]).

Characteristics	Overall (n=136)	High EDRIC (n=68)	Low EDRIC (n=68)	P
Sex(%)				
Female	11 (8.1)	6 (8.8)	5 (7.4)	0.753
Male	125 (91.9)	62 (91.2)	63 (92.6)	
ECOG(%)				
<1	14 (10.3)	7 (10.3)	7 (10.3)	1.000
0-1	122 (89.7)	61 (89.7)	61 (89.7)	
Age (%)				
<65	73 (53.7)	35 (51.5)	38 (55.9)	0.606
≥65	63 (46.3)	33 (48.5)	30 (44.1)	
TNM stage(%)				
IIIA	63 (46.3)	24 (35.3)	39 (57.3)	0.010
IIIB	53 (39.0)	35 (51.5)	18 (26.5)	
IIIC	20 (14.7)	9 (13.2)	11 (16.2)	
Smoking (%)				
No	19 (14.0)	9 (13.2)	10 (14.7)	0.805
Yes	117 (86.0)	59 (86.8)	58 (85.3)	
Pattern of CRT(%)				
cCRT	40 (29.4)	24 (35.3)	16 (23.5)	0.132
sCRT	96 (70.6)	44 (64.7)	52 (76.5)	
Total chemotherapy cycles (%)				
≤6	96 (70.6)	52 (76.5)	44 (64.7)	0.132
>6	40 (29.4)	16 (23.5)	24 (35.3)	
Total immunotherapy cycles (%)				
≤6	97 (71.3)	51 (75.0)	46 (67.6)	0.343
>6	39 (28.7)	17 (25.0)	22 (32.4)	
Treatment sequencing(%)				
Con	71 (52.2)	41 (60.3)	30 (44.1)	0.157
Ind	45 (33.1)	18 (26.5)	27 (39.7)	
Ind+Con	20 (14.7)	9 (13.2)	11 (16.2)	
Baseline ALC (mean ± SD)	1.80 ± 0.56	1.78 ± 0.53	1.83 ± 0.59	0.616
GC(%)				
No	101 (74.3)	52 (76.5)	49 (72.1)	0.556
Yes	35 (25.7)	16 (23.5)	19 (27.9)	

ECOG, Eastern Cooperative Oncology Group; cCRT, concurrent chemoradiotherapy; sCRT, sequential chemoradiotherapy; Con, consolidation immunotherapy; Ind: Induction chemoimmunotherapy; Ind+Con, Induction chemoimmunotherapy followed by consolidation immunotherapy; GC, glucocorticoid; mean (SD), Quantitative data were shown as mean (SD).

**Figure 1:**
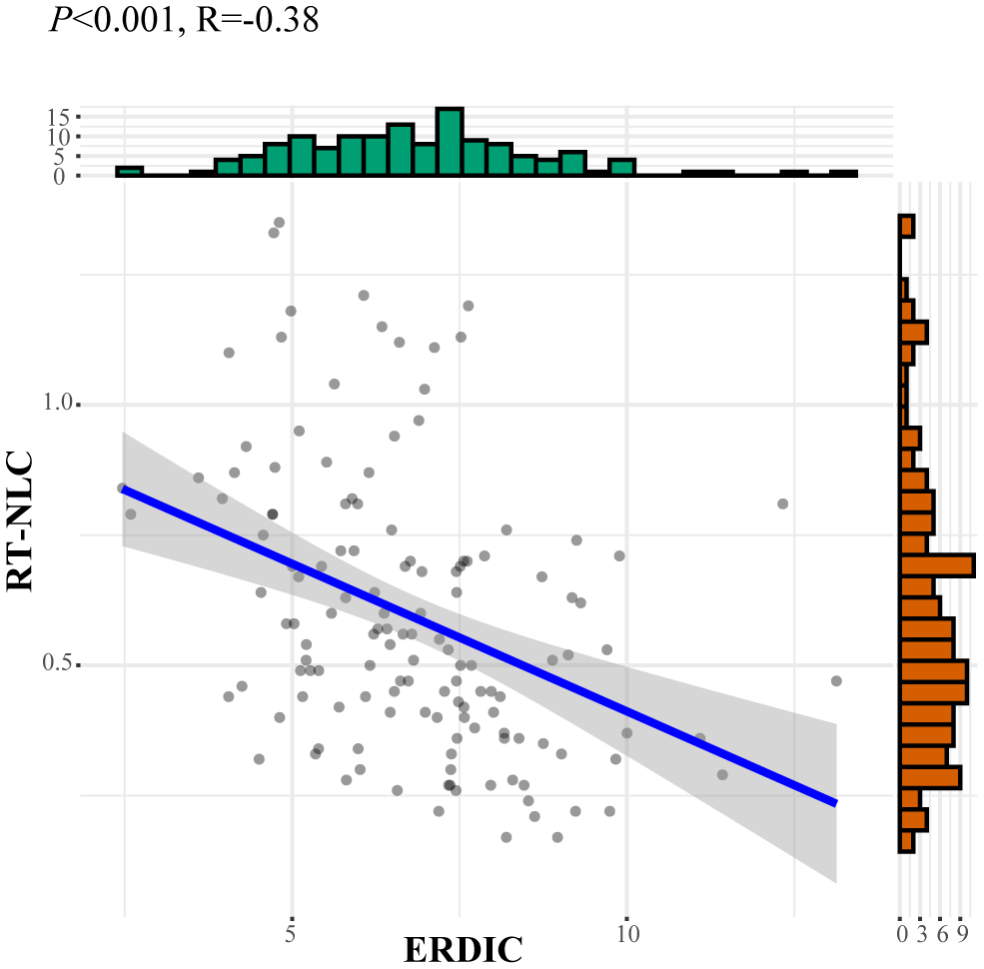
Correlation between EDRIC and RT-NLC. The X-axis shows EDRIC values, and the Y-axis shows RT-NLC levels. Gray dots represent individual patients, overlaid with a fitted regression line and a shaded 95% confidence band. The top histogram illustrates the distribution of EDRIC values, while the right histogram depicts the frequency distribution of RT-NLC values.

The median EDRIC was 6.75 Gy (range, 2.47–13.13). A significant negative correlation was observed between EDRIC and RT-NLC (r = –0.38, *P* < 0.001), indicating that higher radiation exposure of circulating immune cells was associated with more severe treatment-related lymphopenia (**Figure 1**).

Compared with patients with high EDRIC and low RT-NLC (Group 1), those with high EDRIC and high RT-NLC (Group 2) demonstrated a trend toward improved overall survival (OS) (not reached vs. 32.4 months, *P* = 0.051). Patients with low EDRIC and low RT-NLC (Group 3) showed significantly prolonged OS (not reached vs. 32.4 months, *P* = 0.009). Similarly, patients with low EDRIC and high RT-NLC (Group 4) exhibited improved OS (49.7 vs. 32.4 months, *P* = 0.012) (**Supplementary Figure 1**).

**Figure 2:**
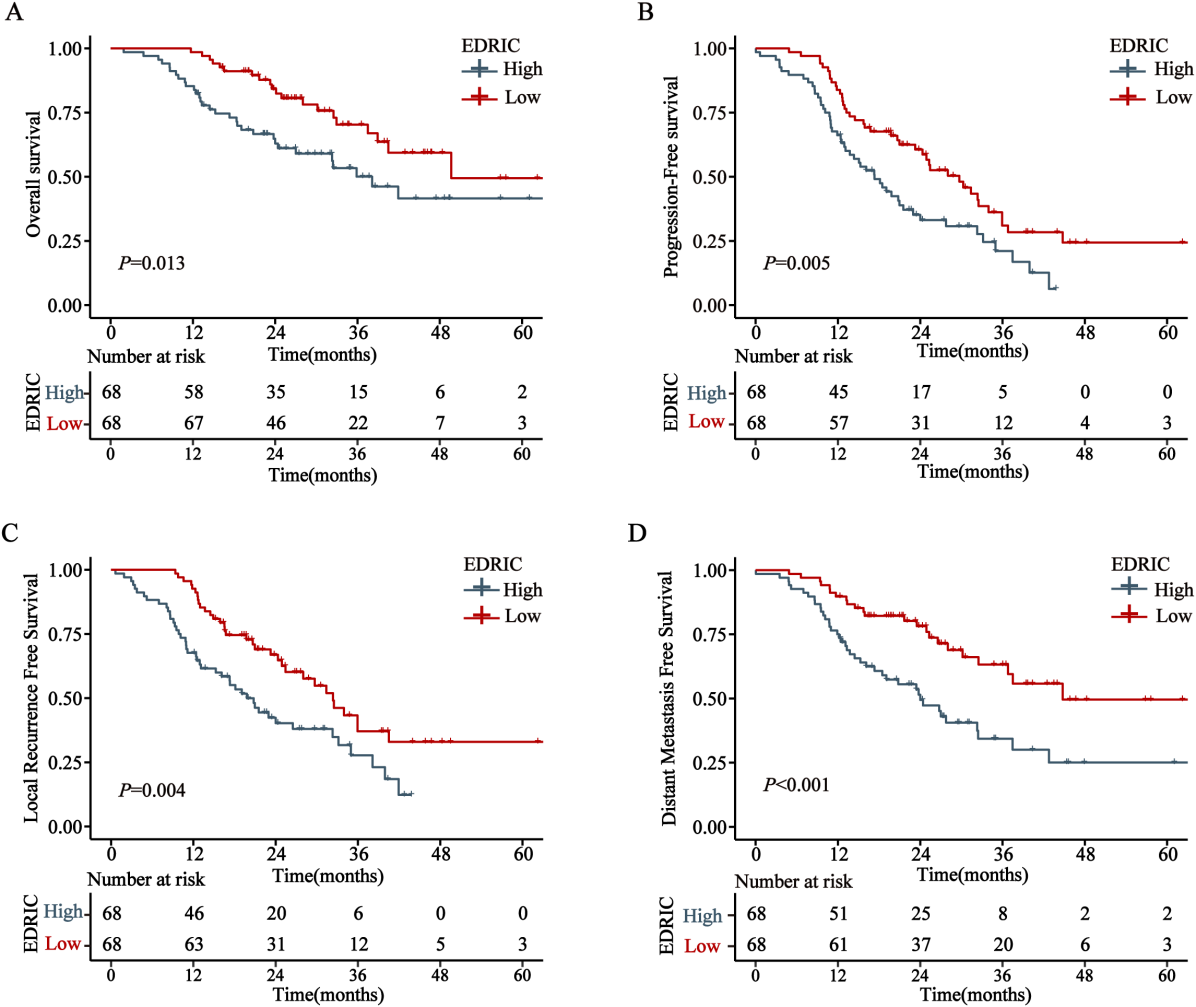
Kaplan–Meier Survival Curves Stratified by EDRIC. **(A)** Overall survival (OS); **(B)** Progression-free survival (PFS); **(C)** Local Recurrence-Free Survival (LRFS); **(D)** Distant metastasis-free survival (DMFS).

**Figure 3:**
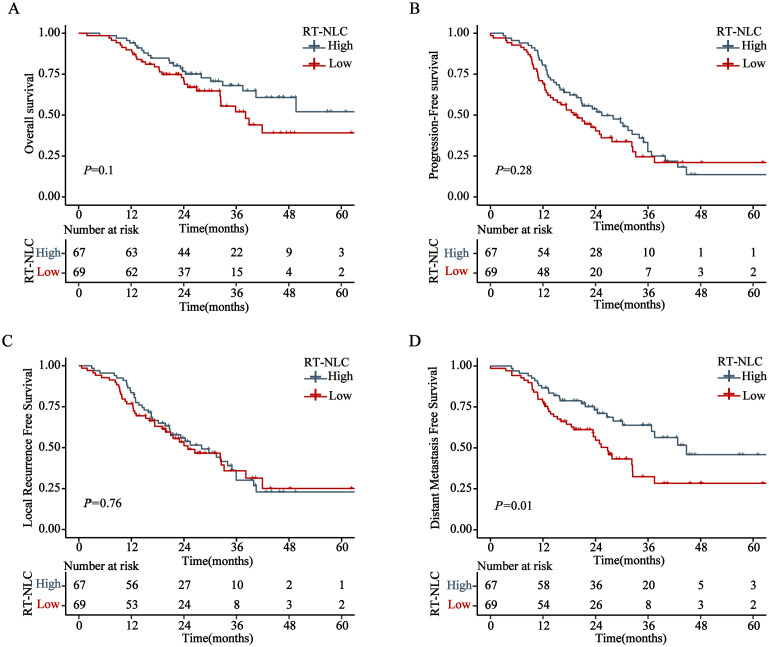
Kaplan–Meier Survival Curves Stratified by RT-NLC. **(A)** OS; **(B)** PFS; **(C)** LRFS; **(D)** DMFS.

With the median follow-up of 49.7 months, patients in the low-EDRIC group (<6.75 Gy) exhibited significantly longer median OS (49.7 vs. 38.1 months, *P* = 0.015), PFS (29.7 vs. 17.3 months, *P* = 0.006), LRFS (32.4 vs. 19.8 months, *P* = 0.004), and DMFS (44.8 vs. 24.0 months, *P* = 0.001) compared to the high-EDRIC group (**Figures 2A–D**). On multivariable analysis, low EDRIC remained an independent predictor of improved OS (HR = 0.51, *P* = 0.020), PFS (HR = 0.57, *P* = 0.008), LRFS (HR = 0.51, *P* = 0.004), and DMFS (HR = 0.42, *P* = 0.001). Additionally, receipt of more than six cycles of immunotherapy was independently associated with better OS (HR = 0.29, *P* = 0.003), PFS (HR = 0.53, *P* = 0.012), LRFS (HR = 0.57, *P* = 0.049), and DMFS (HR = 0.38, *P* = 0.003) (**Supplementary Table 1**, **S2**). Receipt of corticosteroids was associated with longer LRFS(HR = 0.47, *P* = 0.014) (**Supplementary Table 2**). When stratified by RT-NLC, the high RT-NLC group (≥0.54×10^9^/L) showed significantly longer median DMFS (44.8 vs 26.8 months, *P* = 0.012). However, no significant differences were observed in OS, PFS, or LRFS between the groups (**Figures 3A–D**). Multivariable Cox analysis confirmed that more than six cycles of immunotherapy was independently associated with improved OS (HR = 0.29, *P* = 0.003), PFS (HR = 0.52, *P* = 0.011), LRFS (HR = 0.56, *P* = 0.039), and DMFS (HR = 0.41, *P* = 0.008) (**Supplementary Table 3**, **S4**). Receipt of corticosteroids was associated with longer LRFS(HR = 0.49, *P* = 0.020) (**Supplementary Table 4**).

**Figure 4:**
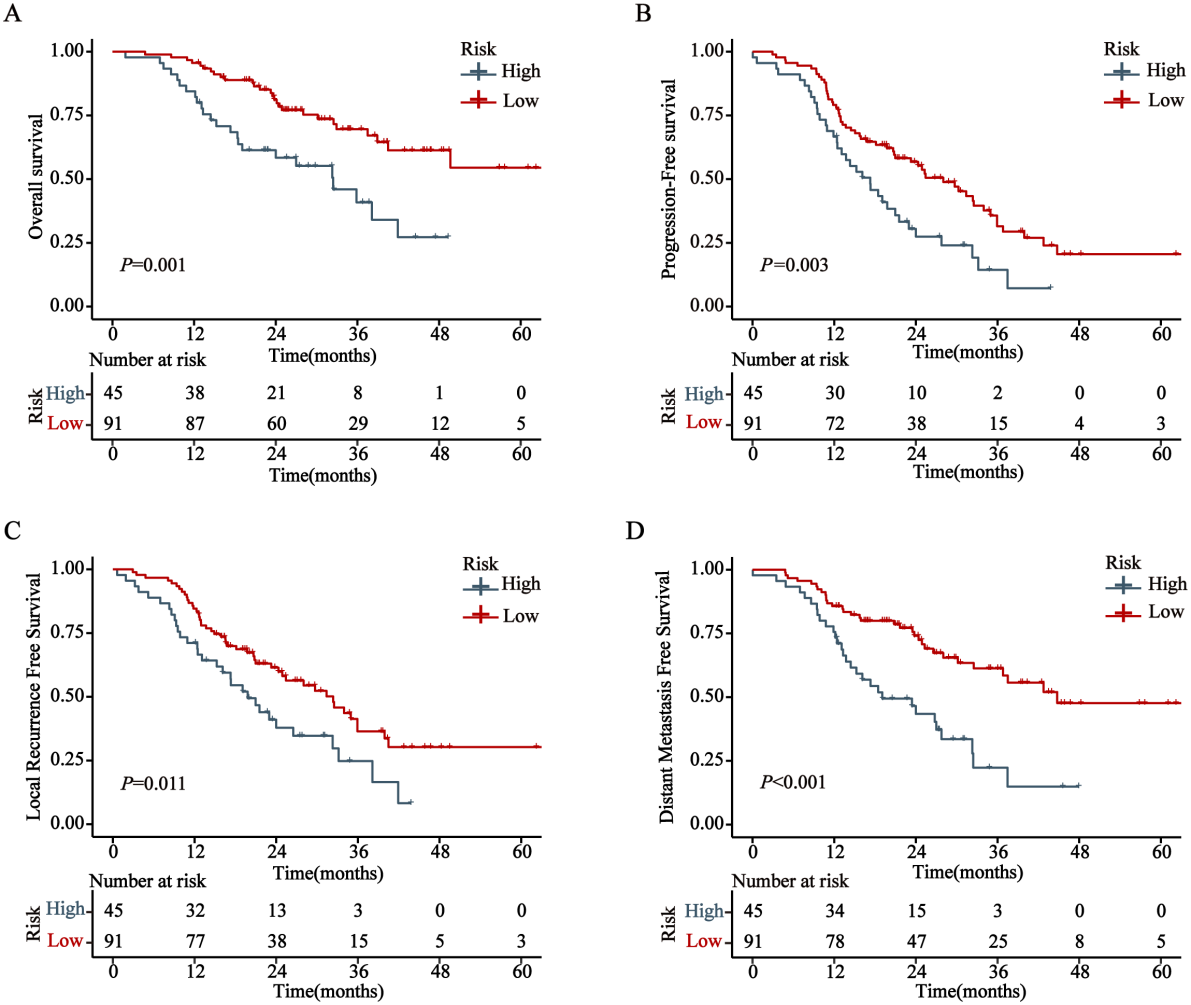
Kaplan–Meier Survival Curves According to Risk Stratification by EDRIC and RT-NLC. **(A)** OS; **(B)** PFS; **(C)** LRFS; **(D)** DMFS.

**Figure 5:**
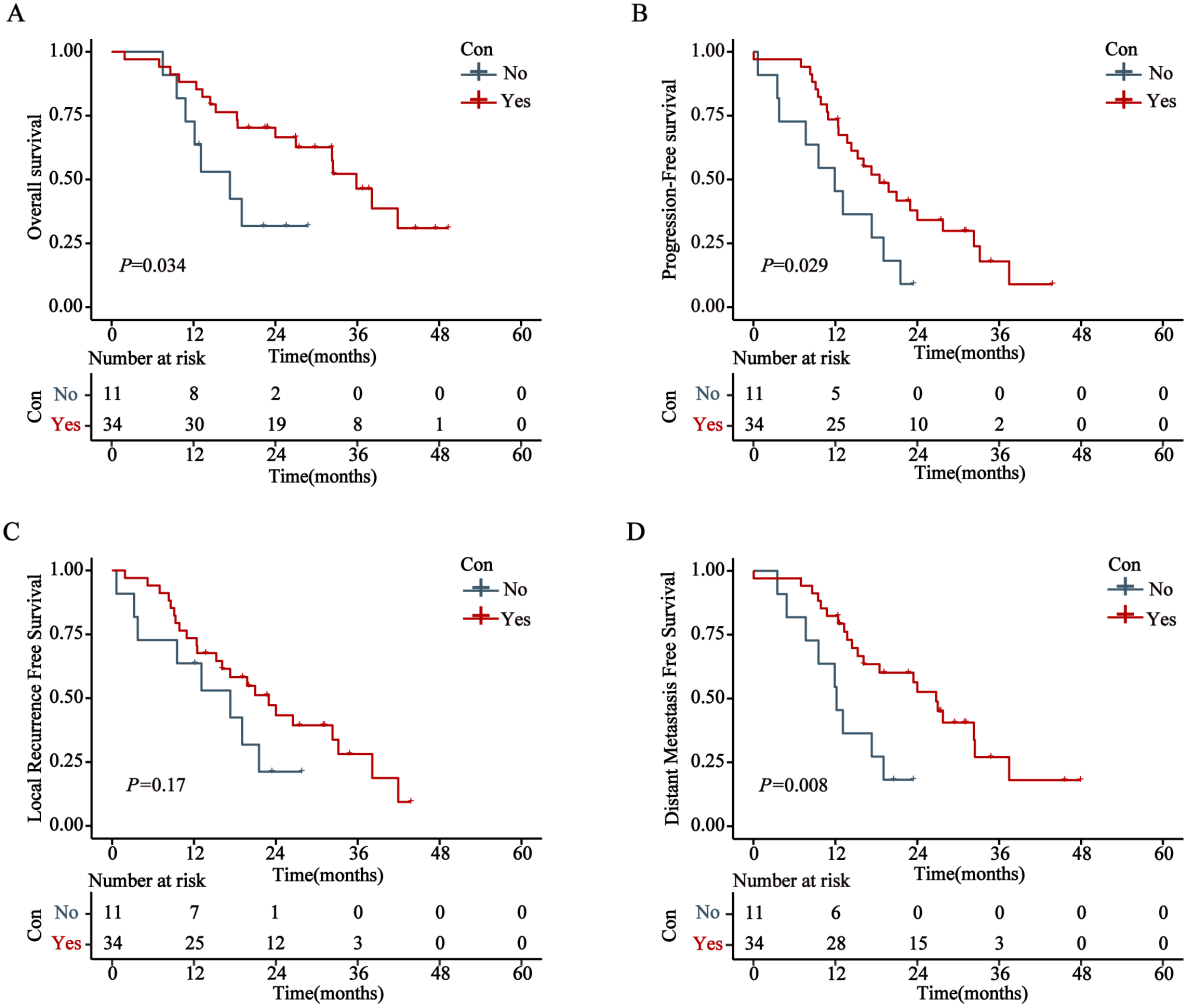
Kaplan–Meier Survival Curves Stratified by Consolidation Immunotherapy in the High-Risk Group. **(A)** OS, **(B)** PFS, **(C)** LRFS, and **(D)** DMFS. Abbreviations: Con:consolidation immunotherapy.

**Figure 6:**
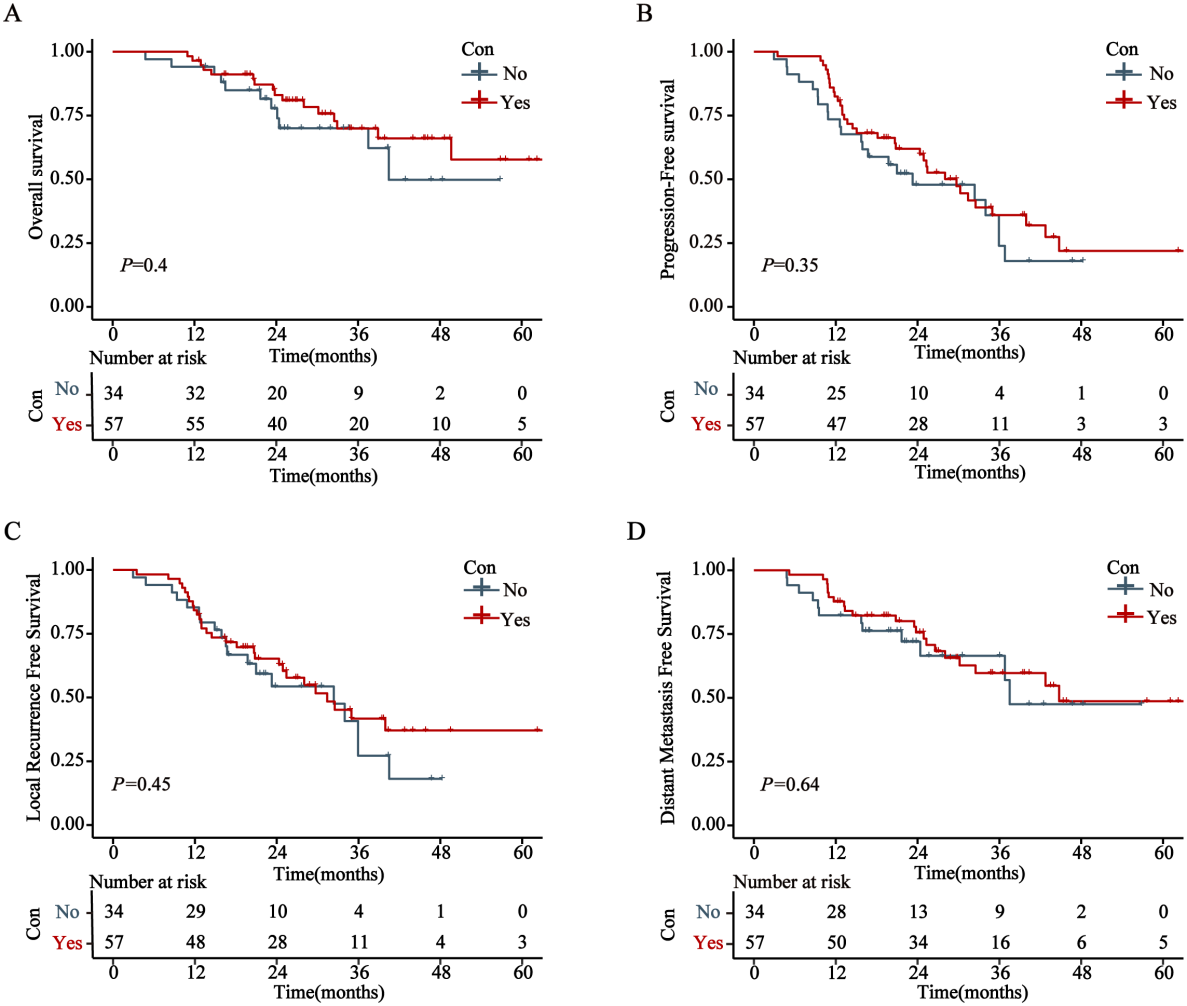
Kaplan–Meier Survival Curves Stratified by Consolidation Immunotherapy in the Low-Risk Group. **(A)** OS, **(B)** PFS, **(C)** LRFS, and **(D)** DMFS. Abbreviations: Con:consolidation immunotherapy.

Patients were classified into high-risk (EDRIC ≥ 6.75 Gy and RT-NLC < 0.54×10^9^/L) and low-risk groups (all others). The low-risk group demonstrated significantly better survival across all endpoints: median OS was not reached vs. 32.4 months (*P* = 0.001), PFS was 28.0 vs. 17.3 months (*P* = 0.004), LRFS was 32.4 vs. 19.8 months (*P* = 0.013), and DMFS was 44.8 vs. 19.1 months (*P* < 0.001) (**Figures 4A–D**). Multivariable analysis identified low-risk status as an independent favorable prognostic factor for OS (HR = 0.44, *P* = 0.005), PFS (HR = 0.59, *P* = 0.020), LRFS (HR = 0.63, *P* = 0.050), and DMFS (HR = 0.40, *P* < 0.001). Furthermore, more than six cycles of immunotherapy remained significantly associated with improved survival across all endpoints (*P<*0.05) (**Supplementary Table 5**, **S6**). Receipt of corticosteroids was associated with longer LRFS (HR = 0.50, *P* = 0.022) (**Supplementary Table 6**). Among high-risk patients, those receiving consolidation immunotherapy showed significantly improved median OS (35.9 vs. 17.3 months, HR = 0.37, *P* = 0.041), PFS (18.5 vs. 11.9 months, HR = 0.44, *P* = 0.034), and DMFS (26.8 vs. 12.2 months, HR = 0.33, *P* = 0.011). However, no significant difference was observed in LRFS (*P* = 0.173) (**Figures 5A–D**). In contrast, consolidation immunotherapy did not significantly improve survival outcomes in the low-risk group (*P*>0.05) (**Figures 6A–D**).

Subgroup analyses demonstrated that within the EDRIC stratification, poorer survival outcomes were consistently observed in the high-EDRIC group compared with the low-EDRIC group across multiple subgroups, including males, ECOG 0–1, age ≥65 years, smokers, patients receiving ≤6 cycles of chemotherapy and immunotherapy, those treated with induction chemoimmunotherapy, and those not receiving corticosteroids (*P* < 0.05) (**Supplementary Figure 2**). In the RT-NLC analysis, a generally consistent trend was identified across most subgroups. Notably, within the concurrent chemoradiotherapy (cCRT) subgroup, improved survival outcomes were observed in the high ALC group compared with the low ALC group (*P* < 0.05) (**Supplementary Figure 3**). Subgroup analyses based on risk stratification further showed that, among patients with ECOG 0–1, those receiving consolidation immunotherapy and those not treated with corticosteroids, the high-risk group exhibited significantly poorer survival outcomes than the low-risk group (*P* < 0.05) (**Supplementary Figure 4**).

After adjustment for TNM stage, EDRIC status remained an independent prognostic factor across all four endpoints: OS (HR = 0.55, 95% CI: 0.31–0.98, *P* = 0.041), PFS (HR = 0.57, 95% CI: 0.37–0.89, *P* = 0.013), LRFS (HR = 0.48, 95% CI: 0.30–0.76, P = 0.002), and DMFS (HR = 0.49, 95% CI: 0.29–0.84, *P* = 0.009) (**Supplementary Table 7**). Low EDRIC was consistently identified as a protective, independent prognostic factor across all endpoints. In addition, stratified survival analyses were conducted according to TNM stage (IIIA, IIIB, and IIIC). A clear stage-dependent prognostic effect of EDRIC was observed, with progressively greater survival separation between EDRIC subgroups as tumor stage advanced. The prognostic impact appeared to be most pronounced in patients with stage IIIC disease (**Supplementary Figure 5**, **S6**).

Landmark analysis, using a 6-month time point to minimize bias, showed no significant difference in survival between groups in the high-risk population in the unadjusted analysis (*P* = 0.569). However, after applying the 6-month landmark adjustment, consolidation immunotherapy was significantly associated with improved survival (*P* = 0.019). In the low-risk population, no significant differences in survival were observed between groups either before or after landmark adjustment (*P* > 0.05). Furthermore, the 12-month landmark sensitivity analysis yielded results consistent with those of the 6-month analysis, further supporting the robustness of these findings (**Supplementary Figure 7**).

## 4 Discussion

This study demonstrated that both EDRIC and the RT-NLC serve as significant prognostic indicators in patients with stage III unresectable NSCLC treated with definitive CRT followed by immunotherapy. Furthermore, a risk stratification model integrating these two parameters effectively identified a high-risk subgroup that derived substantial survival benefit from consolidation immunotherapy. These findings provide valuable insights for individualized treatment strategies in this patient population. Our results align with previous studies that have established the prognostic value of EDRIC in the era of immunotherapy (9–11, 17–20). We confirmed that higher EDRIC is associated with poorer OS, PFS, LRFS, and DMFS, underscoring the dual role of radiotherapy: while directly targeting tumor cells, it may also cause systemic immune suppression through unintended irradiation of circulating lymphocytes. The significant inverse correlation between EDRIC and RT-NLC (r = –0.38, *P* < 0.001) reinforces the biological plausibility of EDRIC as a predictor of treatment-related lymphopenia, consistent with observations in esophageal cancer (21). This correlation likely reflects the dose-dependent depletion of radiosensitive lymphocytes in both lymphoid organs and the circulating pool (22–24).

Notably, low RT-NLC was independently associated only with reduced DMFS, a finding corroborated by studies in nasopharyngeal carcinoma (25). This suggests that severe lymphopenia during treatment may specifically impair the immune system’s ability to control micrometastatic disease, thereby increasing the risk of distant metastasis (26). Several mechanisms may underlie this phenomenon: depletion of circulating lymphocytes may compromise immune surveillance (27, 28); loss of regulatory T-cell function could disrupt microenvironmental control of metastasis (29, 30); and radiation-induced secretion of factors such as Galectin-1 may promote T-cell apoptosis and facilitate metastatic spread (31, 32).

A key finding of our study is that high-risk patients, defined by high EDRIC (≥ 6.75 Gy) and low RT-NLC (< 0.54×10^9^/L), exhibited the poorest baseline outcomes but derived the greatest absolute benefit from consolidation immunotherapy. We hypothesize that although high EDRIC contributes to lymphopenia, it may also enhance immunogenic cell death, promoting the release of tumor antigens and activating innate immune pathways, such as the cGAS–STING axis (33–37) This could enrich the T-cell repertoire for tumor-specific clones. Subsequent ICIs may then reverse the inhibitory state of these primed T cells, enabling robust antitumor responses despite lower lymphocyte counts (36, 38, 39). In contrast, low-risk patients, despite having higher lymphocyte counts, may exhibit weaker radiation-induced immune activation and possess more exhausted or nonspecific T-cell clones, resulting in more modest responses to ICIs (40, 41). This suggests that the quality of immune cells, shaped by effective radiotherapy-induced activation, may be more critical than the absolute quantity of lymphocytes.

EDRIC remained an independent prognostic factor after adjustment for TNM stage, with lower EDRIC consistently associated with improved survival outcomes, in line with prior evidence supporting the clinical relevance of radiation-related immune exposure. Stratified analyses further demonstrated stage-dependent heterogeneity, with a more pronounced prognostic effect observed in advanced disease, particularly in stage IIIC patients, which may be attributable to the increased susceptibility of patients with higher tumor burden to treatment-induced immune impairment. To further examine the stability of this stratification and address potential time-dependent bias, landmark analyses were performed. After adjustment, consolidation immunotherapy was associated with a significant overall survival benefit in the high-risk group, whereas no meaningful benefit was observed in low-risk patients. Although these findings suggest that EDRIC-based stratification may refine patient selection for immunotherapy, they also indicate that unadjusted analyses may underestimate treatment effects due to immortal time bias. Notably, consistent results obtained from both the 6-month and 12-month landmark sensitivity analyses reinforce the robustness of the observed associations, supporting the validity of EDRIC as a clinically informative stratification metric.

In the subgroup analyses, consistent prognostic stratification by EDRIC was observed across different clinical subgroups. In patients who were male, had an ECOG PS of 0–1, were aged ≥65 years, had a history of smoking, or received ≤6 cycles of treatment, high EDRIC was significantly associated with poorer survival outcomes, suggesting that radiotherapy-related immune exposure may exert a sustained impact on prognosis across heterogeneous clinical subgroups. A similar pattern was observed in the RT-NLC analysis. Notably, within the cCRT subgroup, higher RT-NLC was significantly associated with improved survival, suggesting that immune reserve may play a more prominent role under intensified treatment. Furthermore, risk stratification based on EDRIC and RT-NLC demonstrated stable discriminatory performance. In patients with ECOG 0–1 and those receiving consolidation immunotherapy, significantly poorer survival outcomes were observed in the high-risk group compared with the low-risk group, indicating that the combined model may provide greater prognostic value than either parameter alone. In addition, within the corticosteroid use subgroup, the above associations appeared more consistent. Given that corticosteroids were primarily administered for the management of treatment-related adverse events, their use may have introduced indication bias and residual confounding. Therefore, these findings should be interpreted with caution and warrant further validation.

Several limitations should be considered. First, the single-center retrospective design is susceptible to selection bias. Second, the EDRIC model does not account for interpatient variations in bone marrow distribution, lymphocyte subpopulation radiosensitivity, or the potential impact of immune-related adverse events on outcomes. Third, the sample size, particularly within the high-risk subgroup, may limit statistical power. Finally, unmeasured confounders such as systemic inflammatory markers, dynamic lymphocyte changes, and PD-L1 expression could influence the model’s predictive accuracy. Future studies should validate these findings in multicenter prospective cohorts. Incorporating molecular biomarkers and dynamic immune parameters could further refine risk stratification. Additionally, exploring EDRIC-based radiotherapy optimization strategies, such as proton therapy or FLASH radiotherapy, may help minimize immune toxicity and improve the therapeutic ratio of combined-modality treatment.

## 5 Conclusion

This study confirms that both EDRIC and RT-NLC are significant prognostic factors in patients with unresectable stage III NSCLC treated with definitive chemoradiotherapy and immunotherapy. The proposed risk stratification model, which integrates these two parameters, effectively identified high-risk patients who are most likely to benefit from consolidation immunotherapy—particularly in controlling distant metastasis. These findings support the adoption of more personalized immunotherapy strategies in this setting, with consolidation immunotherapy recommended for individuals at high risk of recurrence. Further prospective studies are warranted to validate these results and refine the model for clinical application.

## Data Availability

Data will be made available, upon reasonable request, by the corresponding author.
