# Supplementary material for: Risk stratification model based on estimated dose of radiation to immune cells and radiotherapy-related nadir lymphocyte count for predicting the efficacy of consolidation immunotherapy in stage III non-small cell lung cancer
Source: Front Immunol. 2026 Jul 9;17:1734341. doi: 10.3389/fimmu.2026.1734341 (PMC13392429; doi:10.3389/fimmu.2026.1734341)
Supplement: Supplementary file 1 [file SupplementaryFile1.docx]

**1 Supplementary Figure:**


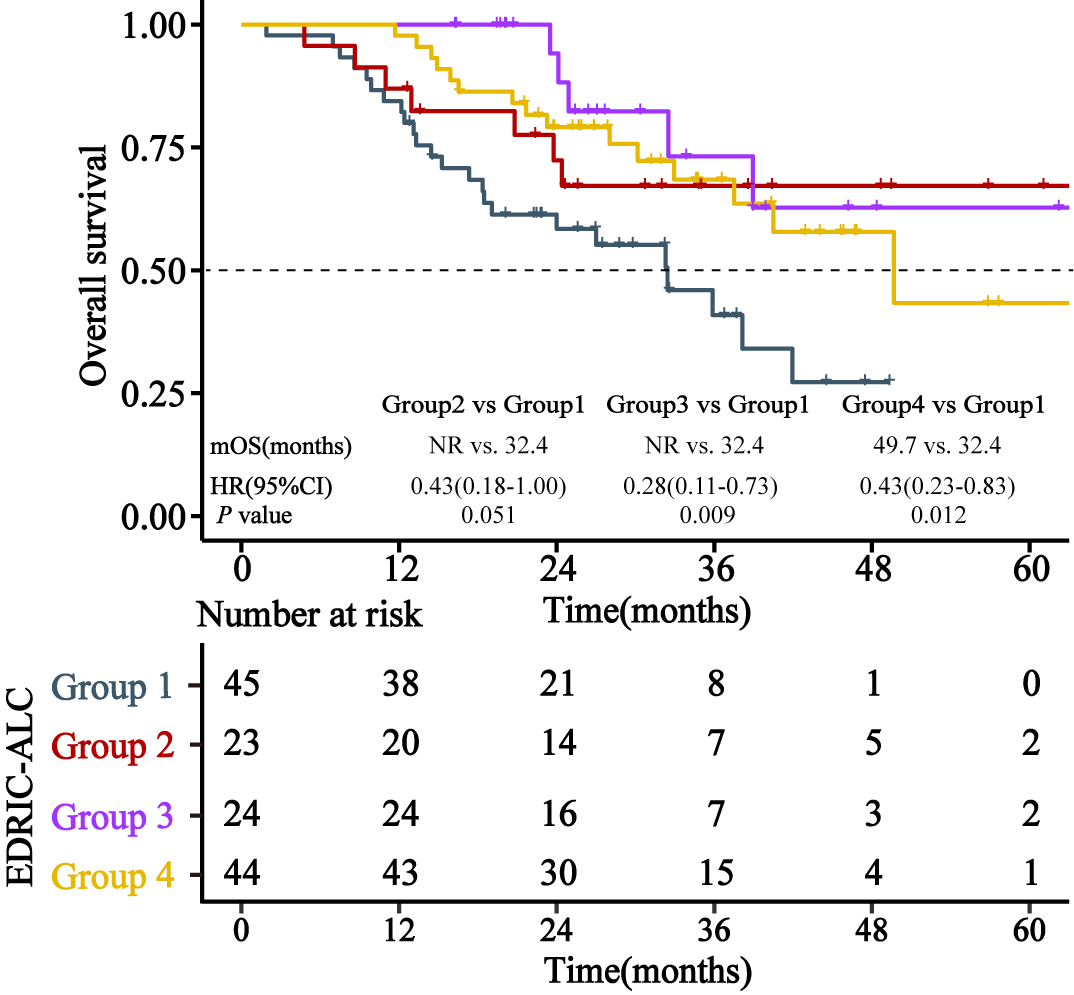


Supplementary Figure 1. Kaplan-Meier survival curves by the EDRIC-ALC stratification.

Patients were stratified into four subgroups based on EDRIC score and absolute lymphocyte count (ALC)

Group 1:High EDRIC-Low ALC; Group 2:High EDRIC-High ALC; Group 3=Low EDRIC-Low ALC; Group 4=Low EDRIC-Hligh ALC

Supplemental Figure 2. EDRIC-stratified subgroup analysis


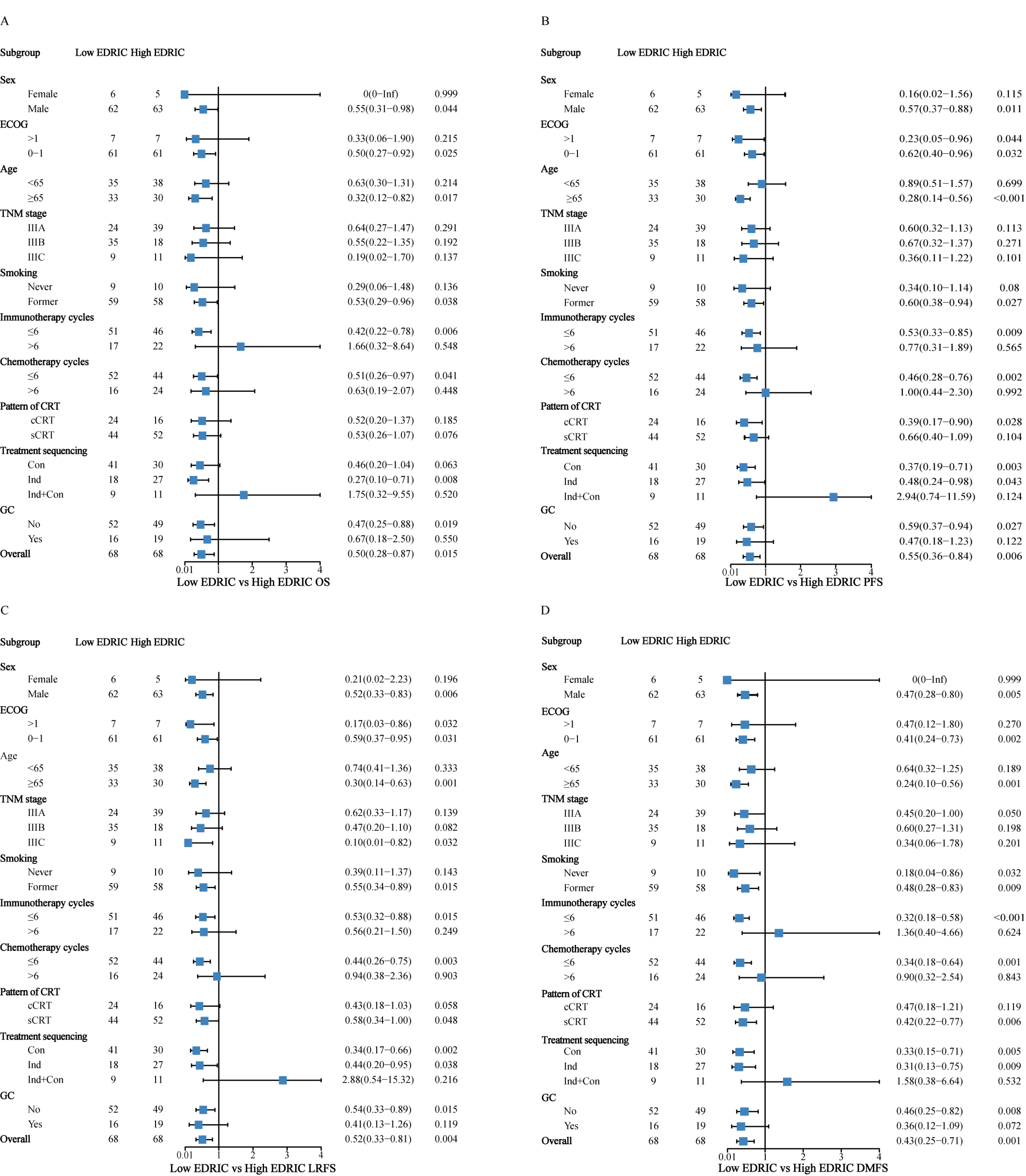


Supplemental Figure 3. ALC-stratified subgroup analysis

Supplemental Figure 4. Risk-stratified subgroup analysis


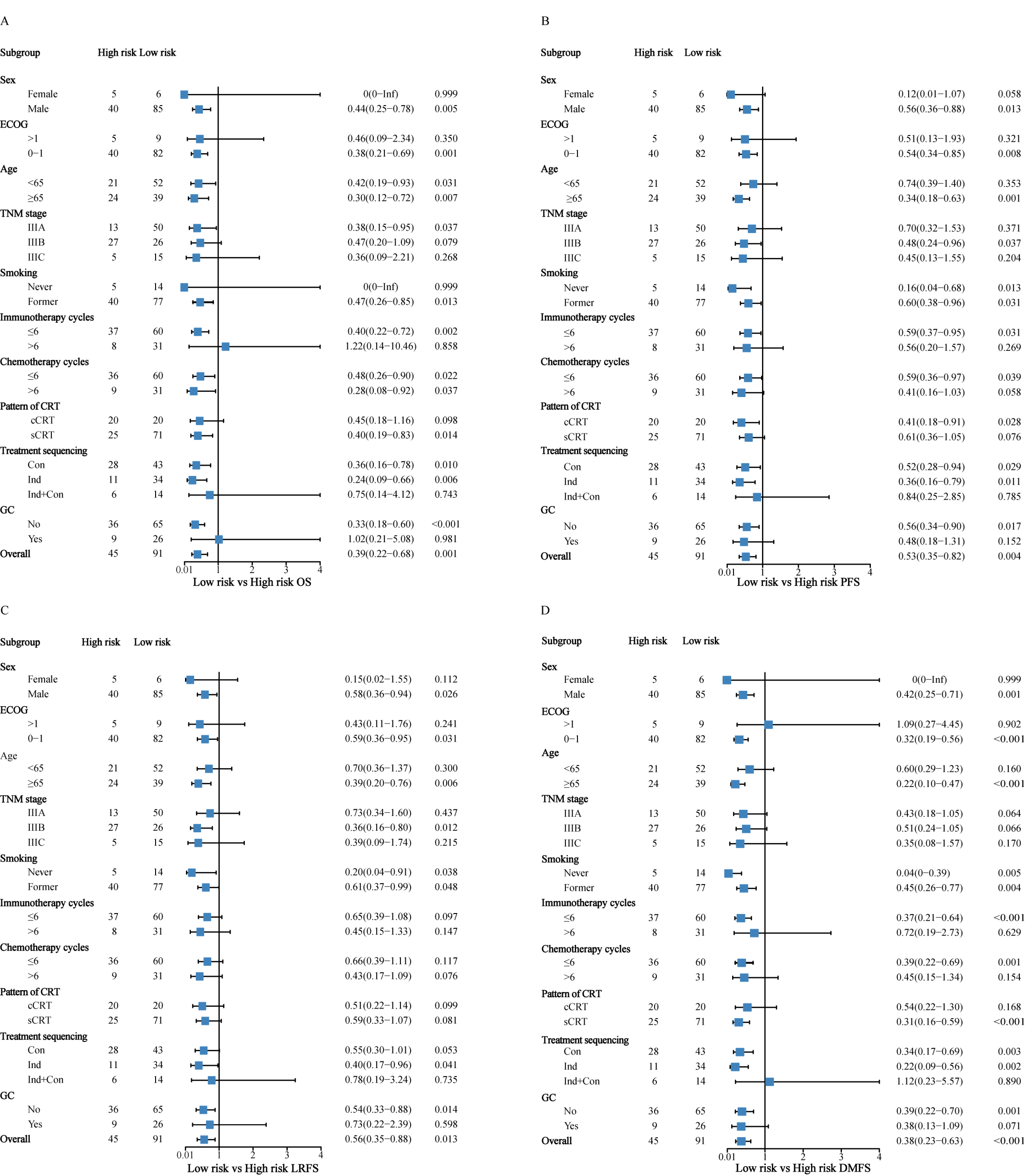


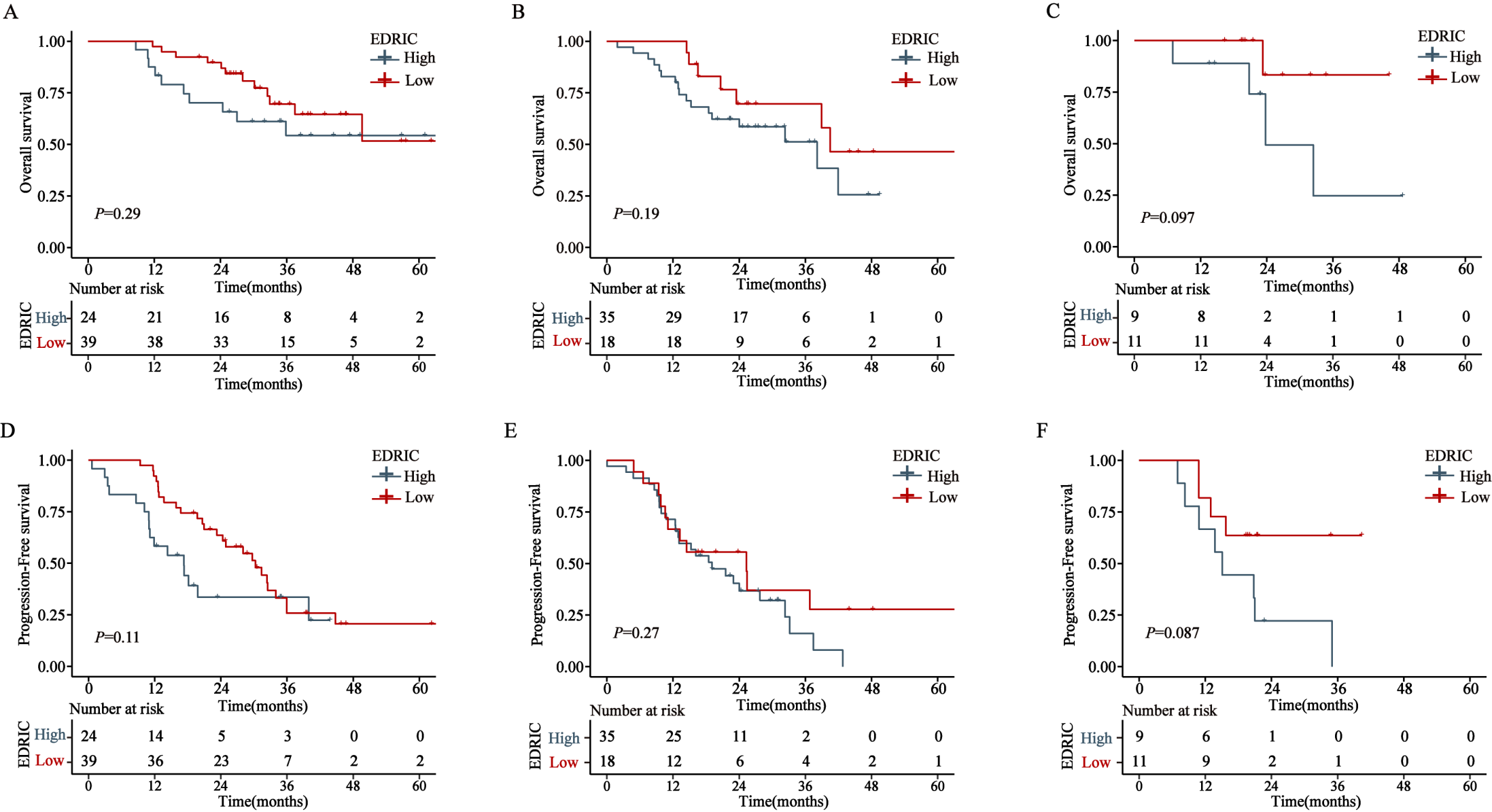
Supplementary Figure 5. Kaplan–Meier curves for OS and PFS according to TNM stage (IIIA–IIIC): (A) IIIA, (B) IIIB, and (C) IIIC.

**
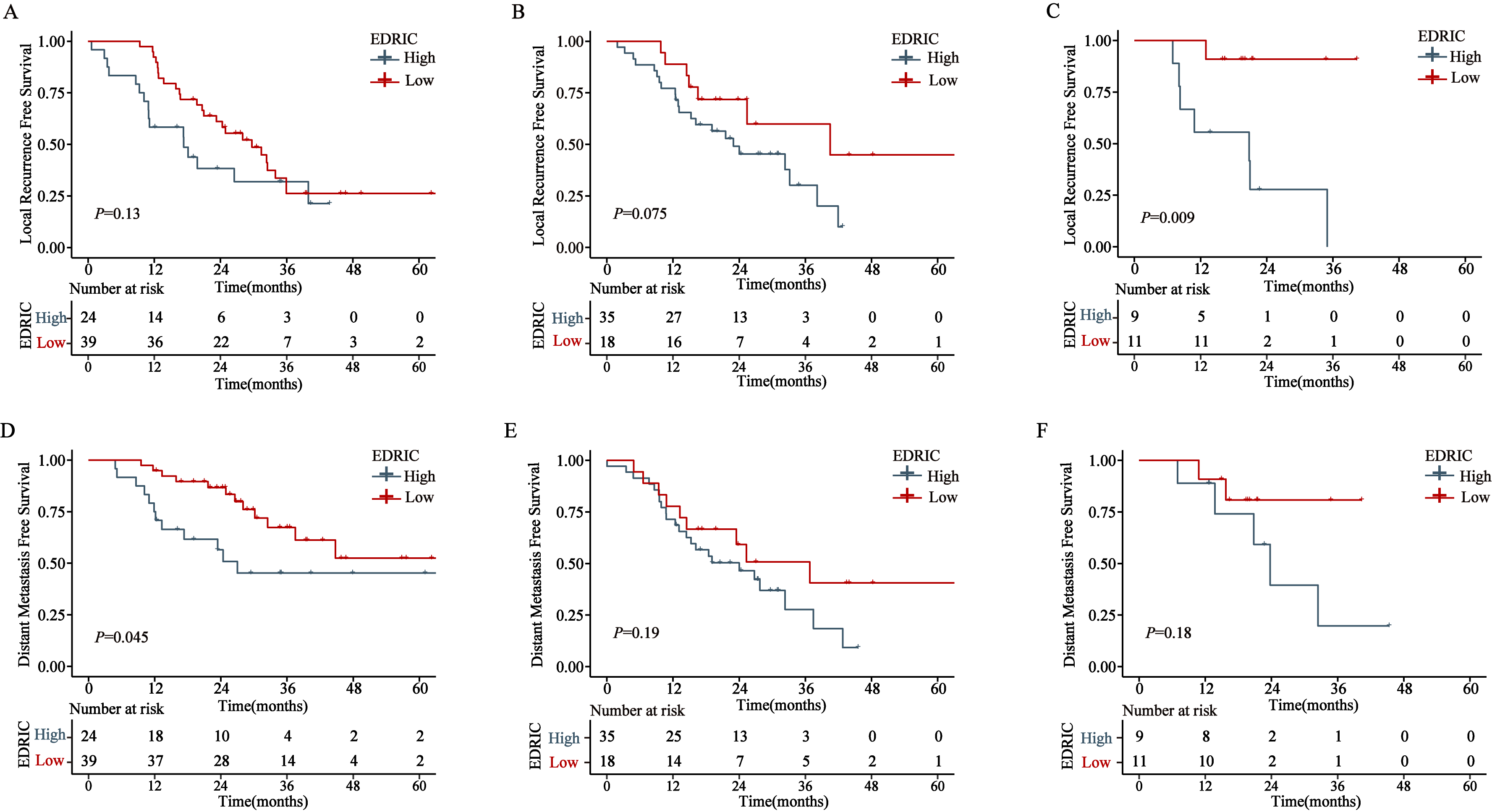
**

Supplementary Figure 6. Kaplan–Meier curves for LRFS and DMFS according to TNM stage (IIIA–IIIC): (A) IIIA, (B) IIIB, and (C) IIIC.


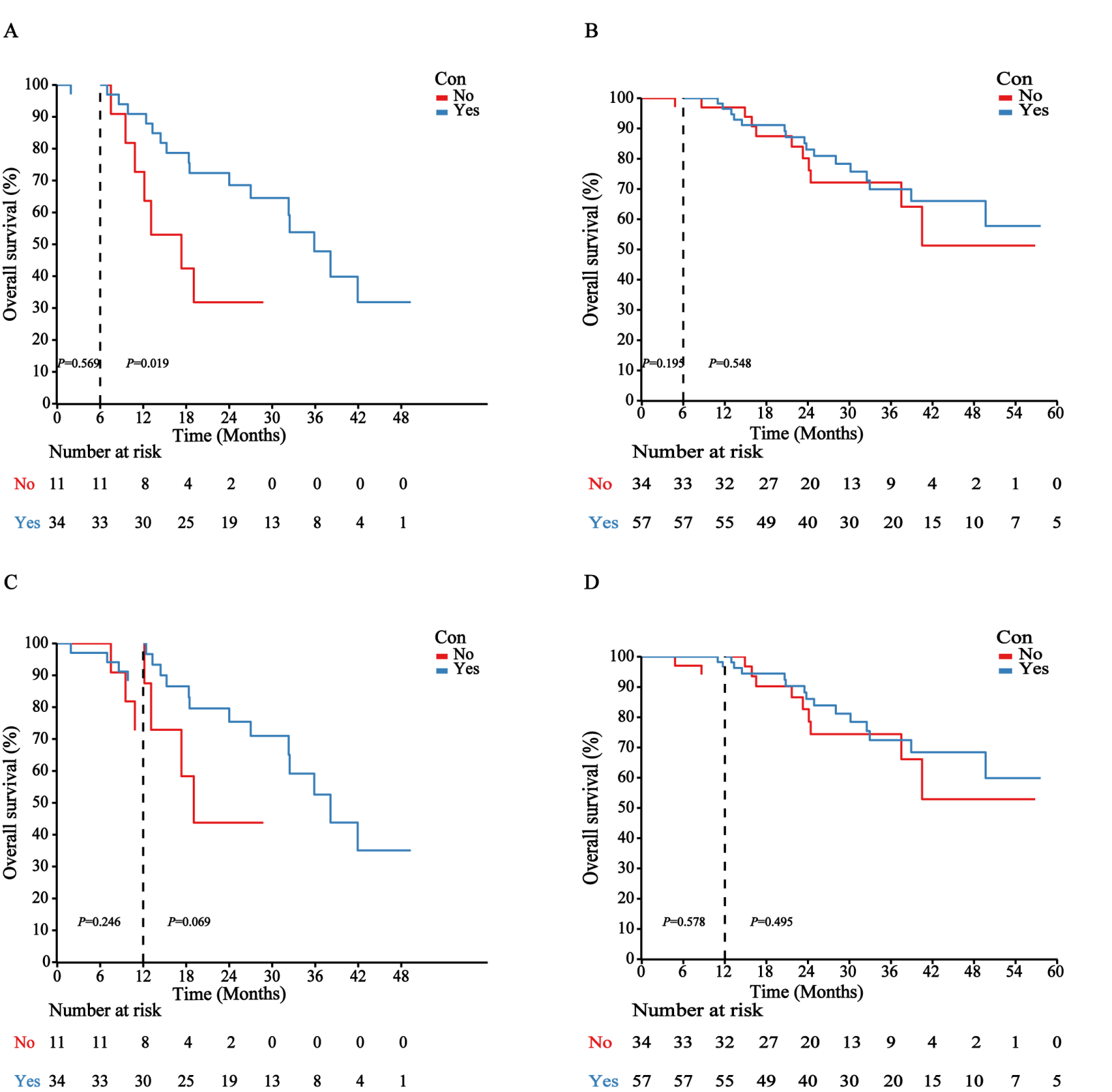


Supplementary Figure 7. Overall survival (OS) according to consolidation immunotherapy across risk groups, based on landmark analyses.

**
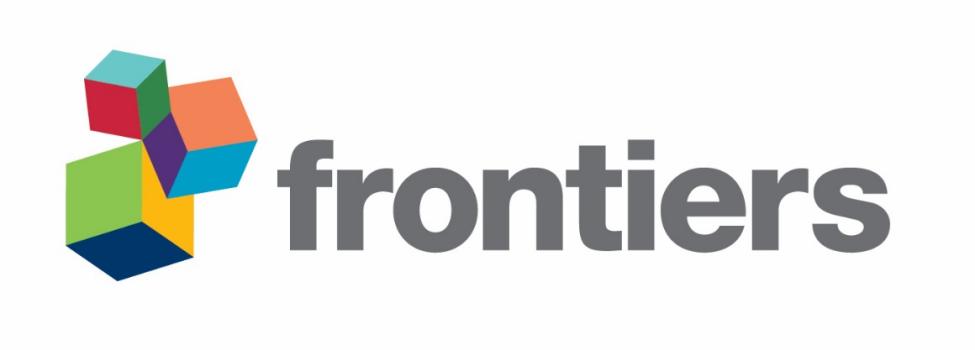
**
