# Supplementary material for: Risk stratification model based on estimated dose of radiation to immune cells and radiotherapy-related nadir lymphocyte count for predicting the efficacy of consolidation immunotherapy in stage III non-small cell lung cancer
Source: Front Immunol. 2026 Jul 9;17:1734341. doi: 10.3389/fimmu.2026.1734341 (PMC13392429; doi:10.3389/fimmu.2026.1734341)
Supplement: Supplementary file 2 [file SupplementaryFile2.docx]

Supplementary Material

# Supplementary Tables

**Table S1 Univariate and multivariate Cox analyses of OS, PFS according to EDRIC groups.**

|  | OS | | | | PFS | | | |
| --- | --- | --- | --- | --- | --- | --- | --- | --- |
| Characteristics | Univariate HR 95%CI | *P* | Multivariate HR 95%CI | *P* | Univariate HR 95%CI | *P* | Multivariate HR 95%CI | *P* |
| Sex |  |  |  |  |  |  |  |  |
| Male vs Female | 0.99(0.31-3.21) | 0.989 | NA | NA | 1.17(0.51-2.68) | 0.718 | NA | NA |
| ECOG |  |  |  |  |  |  |  |  |
| 0-1 vs <1 | 0.80(0.36-1.79) | 0.591 | NA | NA | 1.10(0.57-2.13) | 0.774 | NA | NA |
| Age |  |  |  |  |  |  |  |  |
| ≥65 vs <65 | 0.83(0.48-1.45) | 0.521 | NA | NA | 0.76(0.50-1.15) | 0.194 | NA | NA |
| TNM stage |  |  |  |  |  |  |  |  |
| IIIB vs IIIA | 1.80(1.00-3.23) | 0.048 | NA | NA | 1.34(0.86-2.09) | 0.195 | NA | NA |
| IIIC vs IIIA | 1.07(0.40-2.86) | 0.889 | NA | NA | 1.19(0.62-2.27) | 0.601 | NA | NA |
| Smoking |  |  |  |  |  |  |  |  |
| Yes vs No | 0.96(0.43-2.13) | 0.917 | NA | NA | 1.12(0.61-2.06) | 0.709 | NA | NA |
| Pattern of CRT |  |  |  |  |  |  |  |  |
| sCRT vs cCRT | 0.63(0.36-1.11) | 0.110 | NA | NA | 0.86(0.54-1.34) | 0.497 | NA | NA |
| Total chemotherapy cycles |  |  |  |  |  |  |  |  |
| ＞6 vs ≤6 | 0.56(0.29-1.09) | 0.088 | NA | NA | 0.70(0.44-1.12) | 0.139 | NA | NA |
| Total immunotherapy cycles |  |  |  |  |  |  |  |  |
| ＞6 vs ≤6 | 0.29(0.13-0.64) | 0.002 | 0.29(0.13-0.65) | 0.003 | 0.53(0.32-0.87) | 0.013 | 0.53(0.32-0.87) | 0.012 |
| EDRIC |  |  |  |  |  |  |  |  |
| Low vs High | 0.50(0.28-0.87) | 0.015 | 0.51(0.29-0.89) | 0.020 | 0.55(0.36-0.84) | 0.006 | 0.57(0.37-0.86) | 0.008 |
| Treatment sequencing |  |  |  |  |  |  |  |  |
| Ind vs Con | 1.34(0.74-2.45) | 0.337 | NA | NA | 1.36(0.87-2.14) | 0.179 | NA | NA |
| Ind+Con vs Con | 0.84(0.35-2.04) | 0.705 | NA | NA | 1.10(0.59-2.04) | 0.768 | NA | NA |
| Baseline ALC | 0.94(0.55-1.60) | 0.813 | NA | NA | 1.14(0.78-1.67) | 0.489 | NA | NA |
| GC |  |  |  |  |  |  |  |  |
| Yes vs No | 0.52(0.25-1.06) | 0.074 | 0.50(0.24-1.04) | 0.063 | 0.64(0.38-1.07) | 0.087 | 0.63(0.38-1.06) | 0.081 |

**Abbreviations:** ECOG, Eastern Cooperative Oncology Group; EDRIC, estimated dose of radiation to immune cells; cCRT, concurrent chemoradiotherapy; sCRT, sequential chemoradiotherapy; Con, consolidation immunotherapy; Ind: Induction chemoimmunotherapy ; Ind+Con, Induction chemoimmunotherapy followed by consolidation immunotherapy; GC, glucocorticoid.

**Table S2 Univariate and multivariate Cox analyses of LRFS, DMFS according to EDRIC groups.**

|  | LRFS | | | | DMFS | | | |
| --- | --- | --- | --- | --- | --- | --- | --- | --- |
| Characteristics | Univariate HR 95%CI | *P* | Multivariate HR 95%CI | *P* | Univariate HR 95%CI | *P* | Multivariate HR 95%CI | *P* |
| Sex |  |  |  |  |  |  |  |  |
| Male vs Female | 1.13(0.45-2.82) | 0.790 | NA | NA | 1.15(0.42-3.19) | 0.783 | NA | NA |
| ECOG |  |  |  |  |  |  |  |  |
| 0-1 vs <1 | 1.08(0.52-2.25) | 0.835 | NA | NA | 0.72(0.36-1.47) | 0.370 | NA | NA |
| Age |  |  |  |  |  |  |  |  |
| ≥65 vs <65 | 0.70(0.44-1.11) | 0.129 | NA | NA | 0.87(0.53-1.43) | 0.580 | NA | NA |
| TNM stage |  |  |  |  |  |  |  |  |
| IIIB vs IIIA | 0.93(0.58-1.49) | 0.755 | NA | NA | 2.19(1.28-3.72) | 0.004 | NA | NA |
| IIIC vs IIIA | 0.80(0.37-1.71) | 0.556 | NA | NA | 1.26(0.54-2.93) | 0.598 | NA | NA |
| Smoking |  |  |  |  |  |  |  |  |
| Yes vs No | 0.97(0.51-1.84) | 0.935 | NA | NA | 1.01(0.50-2.05) | 0.977 | NA | NA |
| Pattern of CRT |  |  |  |  |  |  |  |  |
| sCRT vs cCRT | 0.82(0.51-1.32) | 0.414 | NA | NA | 0.72(0.43-1.22) | 0.222 | NA | NA |
| Total chemotherapy cycles |  |  |  |  |  |  |  |  |
| ＞6 vs ≤6 | 0.61(0.36-1.03) | 0.063 | 0.61(0.36-1.04) | 0.069 | 0.63(0.35-1.13) | 0.118 | NA | NA |
| Total immunotherapy cycles |  |  |  |  |  |  |  |  |
| ＞6 vs ≤6 | 0.52(0.30-0.90) | 0.020 | 0.57(0.33-1.00) | 0.049 | 0.38(0.20-0.74) | 0.004 | 0.38(0.20-0.72) | 0.003 |
| EDRIC |  |  |  |  |  |  |  |  |
| Low vs High | 0.52(0.33-0.81) | 0.004 | 0.51(0.32-0.80) | 0.004 | 0.43(0.25-0.71) | 0.001 | 0.42(0.25-0.70) | 0.001 |
| Treatment sequencing |  |  |  |  |  |  |  |  |
| Ind vs Con | 1.22(0.75-1.98) | 0.424 | NA | NA | 1.26(0.73-2.17) | 0.410 | NA | NA |
| Ind+Con vs Con | 0.89(0.43-1.83) | 0.753 | NA | NA | 0.88(0.41-1.90) | 0.745 | NA | NA |
| Baseline ALC | 1.10(0.73-1.67) | 0.646 | NA | NA | 0.93(0.57-1.51) | 0.767 | NA | NA |
| GC |  |  |  |  |  |  |  |  |
| Yes vs No | 0.51(0.28-0.93) | 0.027 | 0.47(0.26-0.86) | 0.014 | 0.82(0.45-1.49) | 0.517 | NA | NA |

**Abbreviations:** ECOG, Eastern Cooperative Oncology Group; EDRIC, estimated dose of radiation to immune cells; cCRT, concurrent chemoradiotherapy; sCRT, sequential chemoradiotherapy; Con, consolidation immunotherapy; Ind: Induction chemoimmunotherapy ; Ind+Con, Induction chemoimmunotherapy followed by consolidation immunotherapy; GC, glucocorticoid.

**Table S3 Univariate and multivariate Cox analyses of OS, PFS according to RT-NLC groups.**

|  | OS | | | | PFS | | | |
| --- | --- | --- | --- | --- | --- | --- | --- | --- |
| Characteristics | Univariate HR 95%CI | *P* | Multivariate HR 95%CI | *P* | Univariate HR 95%CI | *P* | Multivariate HR 95%CI | *P* |
| Sex |  |  |  |  |  |  |  |  |
| Male vs Female | 0.99(0.31-3.21) | 0.989 | NA | NA | 1.17(0.51-2.68) | 0.718 | NA | NA |
| ECOG |  |  |  |  |  |  |  |  |
| 0-1 vs <1 | 0.80(0.36-1.79) | 0.591 | NA | NA | 1.10(0.57-2.13) | 0.774 | NA | NA |
| Age |  |  |  |  |  |  |  |  |
| ≥65 vs <65 | 0.83(0.48-1.45) | 0.521 | NA | NA | 0.76(0.50-1.15 | 0.194 | NA | NA |
| TNM stage |  |  |  |  |  |  |  |  |
| IIIB vs IIIA | 1.80(1.00-3.23) | 0.048 | 1.82(1.01-3.27) | 0.045 | 1.34(0.86-2.09) | 0.195 | NA | NA |
| IIIC vs IIIA | 1.07(0.40-2.86) | 0.889 | 1.24(0.46-3.31) | 0.673 | 1.19(0.62-2.27) | 0.601 | NA | NA |
| Smoking |  |  |  |  |  |  |  |  |
| Yes vs No | 0.96(0.43-2.13) | 0.917 | NA | NA | 1.12(0.61-2.06) | 0.709 | NA | NA |
| Pattern of CRT |  |  |  |  |  |  |  |  |
| sCRT vs cCRT | 0.63(0.36-1.11) | 0.110 | NA | NA | 0.86(0.54-1.34) | 0.497 | NA | NA |
| Total chemotherapy cycles |  |  |  |  |  |  |  |  |
| ＞6 vs ≤6 | 0.56(0.29-1.09) | 0.088 | NA | NA | 0.70(0.44-1.12) | 0.139 | NA | NA |
| Total immunotherapy cycles |  |  |  |  |  |  |  |  |
| ＞6 vs ≤6 | 0.29(0.13-0.64) | 0.002 | 0.29(0.13-0.66) | 0.003 | 0.53(0.32-0.87) | 0.013 | 0.52(0.32-0.86) | 0.011 |
| RT-NLC |  |  |  |  |  |  |  |  |
| Low vs High | 1.58(0.91-2.76) | 0.106 | NA | NA | 1.25(0.83-1.89) | 0.285 | NA | NA |
| Treatment sequencing |  |  |  |  |  |  |  |  |
| Ind vs Con | 1.34(0.74-2.45) | 0.337 | NA | NA | 1.36(0.87-2.14) | 0.179 | NA | NA |
| Ind+Con vs Con | 0.84(0.35-2.04) | 0.705 | NA | NA | 1.10(0.59-2.04) | 0.768 | NA | NA |
| Baseline ALC | 0.94(0.55-1.60) | 0.813 | NA | NA | 1.14(0.78-1.67) | 0.489 | NA | NA |
| GC |  |  |  |  |  |  |  |  |
| Yes vs No | 1.52(0.25-1.06) | 0.074 | 0.46(0.23-0.96) | 0.038 | 0.64(0.38-1.07) | 0.087 | 0.62(0.37-1.04) | 0.071 |

**Abbreviations:** ECOG, Eastern Cooperative Oncology Group; cCRT, concurrent chemoradiotherapy; sCRT, sequential chemoradiotherapy; Con, consolidation immunotherapy; Ind: Induction chemoimmunotherapy ; Ind+Con, Induction chemoimmunotherapy followed by consolidation immunotherapy; GC, glucocorticoid.

**Table S4 Univariate and multivariate Cox analyses of LRFS, DMFS according to RT-NLC groups.**

|  | LRFS | | | | DMFS | | | |
| --- | --- | --- | --- | --- | --- | --- | --- | --- |
| Characteristics | Univariate HR 95%CI | *P* | Multivariate HR 95%CI | *P* | Univariate HR 95%CI | *P* | Multivariate HR 95%CI | *P* |
| Sex |  |  |  |  |  |  |  |  |
| Male vs Female | 1.13(0.45-2.82) | 0.790 | NA | NA | 1.15(0.42-3.19) | 0.783 | NA | NA |
| ECOG |  |  |  |  |  |  |  |  |
| 0-1 vs <1 | 1.08(0.52-2.25) | 0.835 | NA |  | 0.72(0.36-1.47) | 0.370 | NA | NA |
| Age |  |  |  |  |  |  |  |  |
| ≥65 vs <65 | 0.70(0.44-1.11) | 0.129 | NA | NA | 0.87(0.53-1.43) | 0.580 | NA | NA |
| TNM stage |  |  |  |  |  |  |  |  |
| IIIB vs IIIA | 0.93(0.58-1.49) | 0.755 | NA | NA | 2.19(1.28-3.72) | 0.004 | 1.87(1.07-3.25) | 0.028 |
| IIIC vs IIIA | 0.80(0.37-1.70) | 0.556 | NA | NA | 1.26(0.54-2.93) | 0.598 | 1.26(0.54-2.95) | 0.601 |
| Smoking |  |  |  |  |  |  |  |  |
| Yes vs No | 0.97(0.51-1.84) | 0.935 | NA | NA | 1.01(0.50-2.05) | 0.977 | NA | NA |
| Pattern of CRT |  |  |  |  |  |  |  |  |
| sCRT vs cCRT | 0.82(0.51-1.32) | 0.414 | NA | NA | 0.72(0.43-1.22) | 0.222 | NA | NA |
| Total chemotherapy cycles |  |  |  |  |  |  |  |  |
| ＞6 vs ≤6 | 0.61(0.36-1.03) | 0.063 | 0.65(0.38-1.10) | 0.108 | 0.63(0.35-1.13) | 0.118 | NA | NA |
| Total immunotherapy cycles |  |  |  |  |  |  |  |  |
| ＞6 vs ≤6 | 0.52(0.30-0.90) | 0.020 | 0.56(0.32-0.97) | 0.039 | 0.38(0.20-0.74) | 0.004 | 0.41(0.21-0.79) | 0.008 |
| RT-NLC |  |  |  |  |  |  |  |  |
| Low vs High | 1.07(0.69-1.67) | 0.762 | NA | NA | 1.92(1.16-3.20) | 0.012 | 1.50(0.88-2.56) | 0.137 |
| Treatment sequencing |  |  |  |  |  |  |  |  |
| Ind vs Con | 1.22(0.75-1.98) | 0.424 | NA | NA | 1.26(0.73-2.17) | 0.410 | NA | NA |
| Ind+Con vs Con | 0.89(0.43-1.83) | 0.753 | NA | NA | 0.88(0.41-1.90) | 0.745 | NA | NA |
| Baseline ALC | 1.10(0.73-1.67) | 0.646 | NA | NA | 0.93(0.57-1.51) | 0.767 | NA | NA |
| GC |  |  |  |  |  |  |  |  |
| Yes vs No | 0.51(0.28-0.93) | 0.027 | 0.49(0.27-0.89) | 0.020 | 0.82(0.45-1.49) | 0.517 | NA | NA |

**Abbreviations:** ECOG, Eastern Cooperative Oncology Group; cCRT, concurrent chemoradiotherapy; sCRT, sequential chemoradiotherapy; Con, consolidation immunotherapy; Ind: Induction chemoimmunotherapy ; Ind+Con, Induction chemoimmunotherapy followed by consolidation immunotherapy; GC, glucocorticoid.

**Table S5 Univariate and multivariate Cox analyses of OS, PFS according to Risk groups.**

|  | OS | | | | PFS | | | |
| --- | --- | --- | --- | --- | --- | --- | --- | --- |
| Characteristics | Univariate HR 95%CI | *P* | Multivariate HR 95%CI | *P* | Univariate HR 95%CI | *P* | Multivariate HR 95%CI | *P* |
| Sex |  |  |  |  |  |  |  |  |
| Male vs Female | 0.99(0.31-3.21) | 0.989 | NA | NA | 1.17(0.51-2.68) | 0.718 | NA | NA |
| ECOG |  |  |  |  |  |  |  |  |
| 0-1 vs <1 | 0.80(0.36-1.79) | 0.591 | NA | NA | 1.10(0.57-2.13) | 0.774 | NA | NA |
| Age |  |  |  |  |  |  |  |  |
| ≥65 vs <65 | 0.83(0.48-1.45) | 0.521 | NA | NA | 0.76(0.50-1.15) | 0.194 | NA | NA |
| TNM stage |  |  |  |  |  |  |  |  |
| IIIB vs IIIA | 1.80(1.00-3.23) | 0.048 | NA | NA | 1.34(0.86-2.09) | 0.195 | NA | NA |
| IIIC vs IIIA | 1.07(0.40-2.86) | 0.889 | NA | NA | 1.19(0.62-2.27) | 0.601 | NA | NA |
| Smoking |  |  |  |  |  |  |  |  |
| Yes vs No | 0.96(0.43-2.13) | 0.917 | NA | NA | 1.12(0.61-2.06) | 0.709 | NA | NA |
| Pattern of CRT |  |  |  |  |  |  |  |  |
| sCRT vs cCRT | 0.63(0.36-1.11) | 0.110 | NA | NA | 0.86(0.54-1.34) | 0.497 | NA | NA |
| Total chemotherapy cycles |  |  |  |  |  |  |  |  |
| ＞6 vs ≤6 | 0.56(0.29-1.09) | 0.088 | NA | NA | 0.70(0.44-1.12) | 0.139 | NA | NA |
| Total immunotherapy cycles |  |  |  |  |  |  |  |  |
| ＞6 vs ≤6 | 0.29(0.13-0.64) | 0.002 | 0.32(0.14-0.71) | 0.005 | 0.53(0.32-0.87) | 0.013 | 0.57(0.34-0.94) | 0.029 |
| Risk |  |  |  |  |  |  |  |  |
| Low vs High | 0.39(0.22-0.68) | 0.001 | 0.44(0.25-0.78) | 0.005 | 0.53(0.35-0.82) | 0.004 | 0.59(0.38-0.92) | 0.020 |
| Treatment sequencing |  |  |  |  |  |  |  |  |
| Ind vs Con | 1.34(0.74-2.45) | 0.337 | NA | NA | 1.36(0.87-2.14) | 0.179 | NA | NA |
| Ind+Con vs Con | 0.84(0.35-2.04) | 0.705 | NA | NA | 1.10(0.59-2.04) | 0.768 | NA | NA |
| Baseline ALC | 0.94(0.55-1.60) | 0.813 | NA | NA | 1.14(0.78-1.67) | 0.489 | NA | NA |
| GC |  |  |  |  |  |  |  |  |
| Yes vs No | 0.52(0.25-1.06) | 0.074 | 0.52(0.25-1.07) | 0.074 | 0.64(0.38-1.07) | 0.087 | 0.65(0.39-1.10) | 0.108 |

**Abbreviations:** ECOG, Eastern Cooperative Oncology Group; cCRT, concurrent chemoradiotherapy; sCRT, sequential chemoradiotherapy; Con, consolidation immunotherapy; Ind: Induction chemoimmunotherapy ; Ind+Con, Induction chemoimmunotherapy followed by consolidation immunotherapy; GC, glucocorticoid.

**Table S6 Univariate and multivariate Cox analyses of LRFS, DMFS according to Risk groups.**

|  | LRFS | | | | DMFS | | | |
| --- | --- | --- | --- | --- | --- | --- | --- | --- |
| Characteristics | Univariate HR 95%CI | *P* | Multivariate HR 95%CI | *P* | Univariate HR 95%CI | *P* | Multivariate HR 95%CI | *P* |
| Sex |  |  |  |  |  |  |  |  |
| Male vs Female | 1.13(0.45-2.82) | 0.790 | NA | NA | 1.15(0.42-3.19) | 0.783 | NA | NA |
| ECOG |  |  |  |  |  |  |  |  |
| 0-1 vs <1 | 1.08(0.52-2.25) | 0.835 | NA | NA | 0.72(0.36-1.47) | 0.370 | NA | NA |
| Age |  |  |  |  |  |  |  |  |
| ≥65 vs <65 | 0.70(0.44-1.11) | 0.129 | NA | NA | 0.87(0.53-1.43) | 0.580 | NA | NA |
| TNM stage |  |  |  |  |  |  |  |  |
| IIIB vs IIIA | 0.93(0.58-1.49) | 0.755 | NA | NA | 2.19(1.28-3.72) | 0.004 | NA | NA |
| IIIC vs IIIA | 0.80(0.37-1.70) | 0.556 | NA | NA | 1.26(0.54-2.93) | 0.598 | NA | NA |
| Smoking |  |  |  |  |  |  |  |  |
| Yes vs No | 0.97(0.51-1.84) | 0.935 | NA | NA | 1.01(0.50-2.05) | 0.977 | NA | NA |
| Pattern of CRT |  |  |  |  |  |  |  |  |
| sCRT vs cCRT | 0.82(0.51-1.32) | 0.414 | NA | NA | 0.72(0.43-1.22) | 0.222 | NA | NA |
| Total chemotherapy cycles |  |  |  |  |  |  |  |  |
| ＞6 vs ≤6 | 0.61(0.36-1.03) | 0.063 | 0.66(0.39-1.11) | 0.116 | 0.63(0.35-1.13) | 0.118 | NA | NA |
| Total immunotherapy cycles |  |  |  |  |  |  |  |  |
| ＞6 vs ≤6 | 0.52(0.30-0.90) | 0.020 | 0.60(0.34-1.05) | 0.074 | 0.38(0.20-0.74) | 0.004 | 0.41(0.21-0.80) | 0.008 |
| Risk |  |  |  |  |  |  |  |  |
| Low vs High | 0.56(0.35-0.88) | 0.013 | 0.63(0.40-1.00) | 0.050 | 0.38(0.23-0.63) | <0.001 | 0.40(0.24-0.67) | <0.001 |
| Treatment sequencing |  |  |  |  |  |  |  |  |
| Ind vs Con | 1.22(0.75-1.98) | 0.424 | NA | NA | 1.26(0.73-2.17) | 0.410 | NA | NA |
| Ind+Con vs Con | 0.89(0.43-1.83) | 0.753 | NA | NA | 0.88(0.41-1.90) | 0.745 | NA | NA |
| Baseline ALC | 1.10(0.73-1.67) | 0.646 | NA | NA | 0.93(0.57-1.51) | 0.767 | NA | NA |
| GC |  |  |  |  |  |  |  |  |
| Yes vs No | 0.51(0.28-0.93) | 0.027 | 0.50(0.27-0.90) | 0.022 | 0.82(0.45-1.49) | 0.517 | NA | NA |

**Abbreviations:** ECOG, Eastern Cooperative Oncology Group; cCRT, concurrent chemoradiotherapy; sCRT, sequential chemoradiotherapy; Con, consolidation immunotherapy; Ind: Induction chemoimmunotherapy ; Ind+Con, Induction chemoimmunotherapy followed by consolidation immunotherapy; GC, glucocorticoid.

| **Table S7 Multivariable Cox regression results for four survival outcomes adjusted for EDRIC status and TNM stage** | | | |
| --- | --- | --- | --- |
| Outcome | Variables | HR (95%CI) | *P* |
| OS | EDRIC(Low vs High) | 0.55(0.31-0.98) | 0.041 |
|  | TNM stage (IIIB vs IIIA) | 1.57 (0.86-2.86) | 0.142 |
|  | TNM stage (IIIC vs IIIA) | 1.04(0.39-2.77) | 0.943 |
| PFS | EDRIC(Low vs High) | 0.57(0.37 -0.89) | 0.013 |
|  | TNM stage (IIIB vs IIIA) | 1.12 (0.70 -1.78) | 0.641 |
|  | TNM stage (IIIC vs IIIA) | 1.13 (0.59- 2.16) | 0.716 |
| LRFS | EDRIC(Low vs High) | 0.48(0.30-0.76) | 0.002 |
|  | TNM stage (IIIB vs IIIA) | 0.73 (0.45-1.21) | 0.222 |
|  | TNM stage (IIIC vs IIIA) | 0.76(0.35-1.63) | 0.481 |
| DMFS | EDRIC(Low vs High) | 0.49(0.29-0.84) | 0.009 |
|  | TNM stage (IIIB vs IIIA) | 1.79(1.03-3.11) | 0.039 |
|  | TNM stage (IIIC vs IIIA) | 1.14(0.49-2.67) | 0.759 |

**
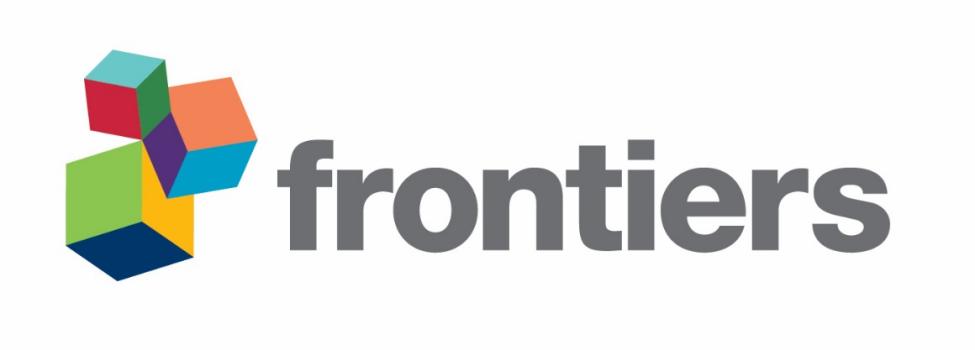
**
